# Vagus Nerve Stimulation Therapy for Drug-Resistant Epilepsy in Children—A Literature Review

**DOI:** 10.3390/jcm13030780

**Published:** 2024-01-29

**Authors:** Mitsumasa Fukuda, Takeshi Matsuo, So Fujimoto, Hirofumi Kashii, Ai Hoshino, Akihiko Ishiyama, Satoko Kumada

**Affiliations:** 1 Department of Neuropediatrics, Tokyo Metropolitan Neurological Hospital, Fuchu 183-0042, Japan; ocean.letter@gmail.com (H.K.); aizm0505@yahoo.co.jp (A.H.); akihiko_ishiyama@tmhp.jp (A.I.); satoko_kumada@tmhp.jp (S.K.); 2 Department of Neurosurgery, Tokyo Metropolitan Neurological Hospital, Fuchu 183-0042, Japan; takeshi_matsuo@tmhp.jp (T.M.); sou_fujimoto@tmhp.jp (S.F.)

**Keywords:** vagus nerve stimulation, VNS, epilepsy, children, quality of life

## Abstract

Vagus nerve stimulation (VNS) is a palliative treatment for drug-resistant epilepsy (DRE) that has been in use for over two decades. VNS suppresses epileptic seizures, prevents emotional disorders, and improves cognitive function and sleep quality, a parallel effect associated with the control of epileptic seizures. The seizure suppression rate with VNS increases monthly to annually, and the incidence of side effects reduces over time. This method is effective in treating DRE in children as well as adults, such as epilepsy associated with tuberous sclerosis, Dravet syndrome, and Lennox–Gastaut syndrome. In children, it has been reported that seizures decreased by >70% approximately 8 years after initiating VNS, and the 50% responder rate was reported to be approximately 70%. VNS regulates stimulation and has multiple useful systems, including self-seizure suppression using magnets, additional stimulation using an automatic seizure detection system, different stimulation settings for day and night, and an automatic stimulation adjustment system that reduces hospital visits. VNS suppresses seizures and has beneficial behavioral effects in children with DRE. This review describes the VNS system, the mechanism of the therapeutic effect, the specific stimulation adjustment method, antiepileptic effects, and other clinical effects in patients with childhood DRE.

## 1. Introduction

Vagus nerve stimulation (VNS) is a palliative therapy that stimulates the vagus nerves. Implantable neurostimulators are an adjunct treatment to drug therapy for drug-resistant epilepsy (DRE) and provide an alternative for patients who are not candidates for resective surgery. Currently, three treatment methods are used in clinical practice: VNS, reactive nerve stimulation, and deep brain stimulation. Among them, VNS is the first neuromodulatory device approved for the treatment of epilepsy. VNS was approved in the United States in 1997, and the number of implementations globally was approximately 125,000 in 2020, according to the manufacturing company. It has also been used in >35,000 pediatric patients worldwide and is approved as a long-term treatment for DRE in children [1].

VNS suppresses epileptic seizures, prevents emotional disorders, and improves cognitive function; further, it has a parallel effect associated with the control of epileptic seizures. In Japan, there are no restrictions for its use related to age or seizure type for children with DRE. Currently, it is an essential treatment method for patients who are resistant to any anti-seizure medication and are unsuitable for open epilepsy surgery such as corpus callosotomy or focal cortical resection or for patients for whom surgery is not sufficiently effective.

This review describes the VNS system, the mechanism of therapeutic effect, the specific stimulation adjustment method, antiepileptic effects, and other clinical effects in patients with childhood DRE.

## 2. VNS Therapy Device

**Figure 1:**
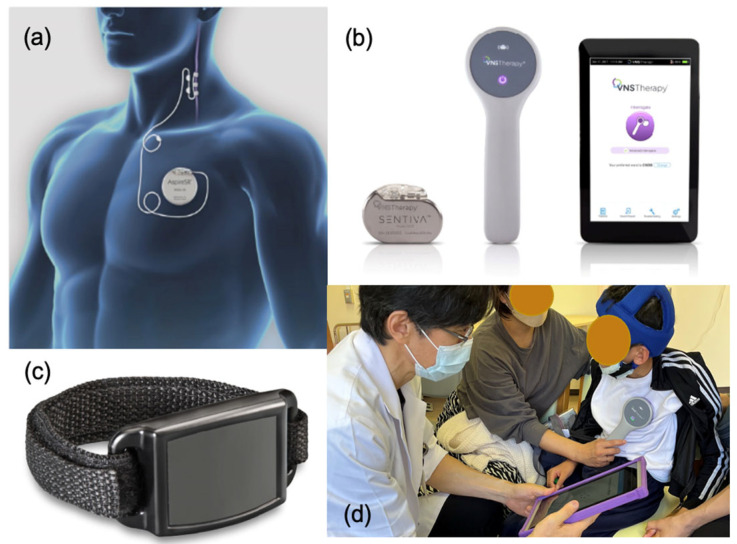
Vagus nerve stimulation (VNS) therapy device.: (**a**) The implanted VNS device consists of a pulse generator (AspireSR Model 106) and a spiral electrode wrap around the left vagus nerve. (**b**) The VNS device comprises a pulse generator (SenTiva Model 1000), a programming wand, and a notepad. (**c**) The magnet is used as an external magnet that can initiate temporary stimulation using a self-adjustment method. (**d**) Parameter setting is facilitated using a programming wand connected to a hand-held computer using radio frequencies. AspireSR and SenTiva are supplied by LivaNova PLC—London, United Kingdom.

The VNS device comprises four devices: (1) An implantable pulse generator; (2) A spiral-implanted electrode; (3) A device that programs the stimulation conditions of the subcutaneously implanted generator from outside the body: (4) An external magnet that can initiate temporary stimulation using a self-adjustment method (Figure 1a–c).

The implantation procedure requires two incisions in the left chest for the generator and in the left neck for the lead. The distal part of the lead is attached to the vagus nerve via spiral tethers, and the other side is inserted subcutaneously into the upper chest using a tunneling tool for connecting to a generator. Anatomically and physiologically, the right vagus nerve mainly transmits downward impulses from the center to the heart’s sinoatrial node. In fact, VNS does affect the heart and is explored as a potential treatment for heart conditions [2]. On the contrary, the left vagus nerve mainly transmits upward impulses from the internal organs to the center rather than to the right [3]. Therefore, a spiral electrode is placed to stimulate the vagus nerve on the left side of the neck, which is unlikely to affect the heart. The stimulation is intermittent, and the parameters are programmable. The parameter setting is facilitated using a programming wand connected to a hand-held computer using radio frequencies (Figure 1d).

## 3. Anatomy of the Vagus Nerve and Mechanism of Antiepileptic Action of VNS

**Figure 2:**
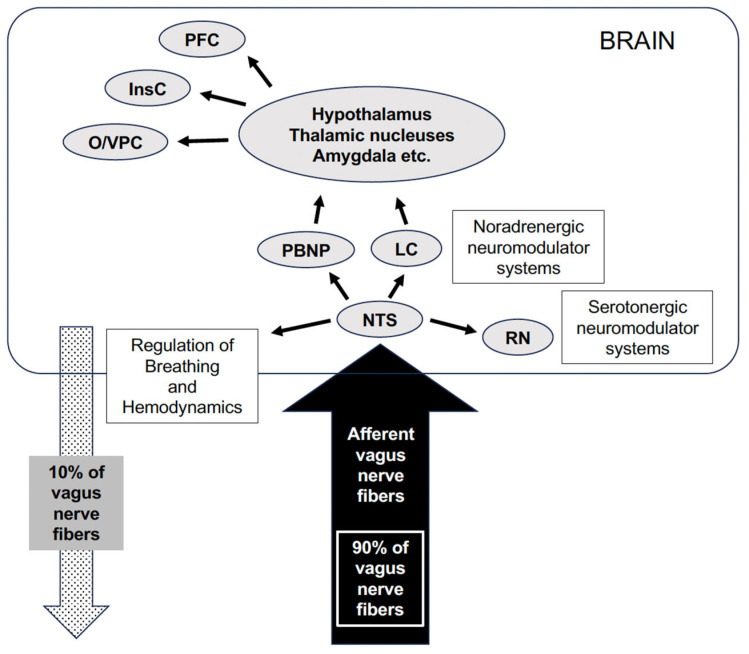
The vagal efferent fibers send the signals “down” from the brain, which account for approximately 10% of all fibers, and the vagal afferents “up” from the intestinal wall to the brain, accounting for 90% of all fibers. Afferent fibers from the NTS project most densely to the PBNP, with the NTS also projecting to noradrenergic (LC) and serotonergic (RN) neuromodulatory systems. Vagal information is relayed to several mostly subcortical structures, including the hypothalamus, the nucleus of the amygdala, the nucleus of the stria terminalis, and the intralaminar thalamic nucleus. Vagal afferent information is also sent to the anterior insular cortex (Ins), which communicates with more rostral regions of the cortex (O/VPC) and PFC. NTS, nucleus tractus solitarius; PBNP, parabrachial nucleus of the pons; LC, locus coeruleus; RN, raphe nucleus; InsC, insular cortex; O/VPC orbital and ventrolateral prefrontal cortex; PFC, prefrontal cortex [8].

The vagus nerve is a complex nerve that is both efferent and afferent; approximately 90% of the nerves are afferent, transmitting information from each thoracic and abdominal organ to the central nervous system. The afferent fibers of the vagus nerve ascend and are relayed to the nucleus tractus solitarius (NTS) on both sides of the medulla oblongata. The output from the NTS is further divided into three routes: (1) Somatic motor nerves of the medulla oblongata (regulating breathing, heart rhythm, and blood pressure); (2) Medullary reticular formation (involved in respiratory reflexes); (3) The third cerebrum pathway is divided into two pathways—one that connects directly to the hypothalamus, amygdala, and limbic system from the NTS and the other that connects mainly to the intralaminal nuclei of the thalamus through the parabrachial nucleus and reaches the cerebral cortex through the midline nuclei [4,5] (Figure 2). The presumed mechanism of VNS therapy is that upward activity induced by VNS broadly affects the limbic system and cortex, suppressing generalized epileptic waves in clinical physiology and local cerebral blood flow in functional imaging [6,7].

Neuroendocrinologically, VNS is thought to cause changes in the gamma-aminobutyric acid (GABA) system nerve activity, norepinephrine system nerve activity, and amino acid metabolism. In addition, basic research on this treatment method has reported increases in GABA in the piriform cortex and prolonged amygdala kindling time [9,10]. Neuroimmunologically, a previous report using pro- and anti-inflammatory cytokines in peripheral blood indicated that VNS causes rebalancing of the immune system, reducing neurotoxins, increasing neuroprotective kynurenine metabolites, and normalizing cortisol levels [11]. Another study using gene expression in peripheral blood mononuclear cells indicated that VNS suppressed epileptic seizures through anti-inflammatory effects [12]. In neurophysiological studies, VNS suppresses seizures by regulating cerebral blood flow and desynchronizing paroxysmal electroencephalogram (EEG) patterns neurophysiologically [7,13]. The effects of VNS appeared slowly over time, suggesting the involvement of multiple mechanisms as described above.

## 4. Specific Basic Stimulus Adjustment of VNS

**Figure 3:**
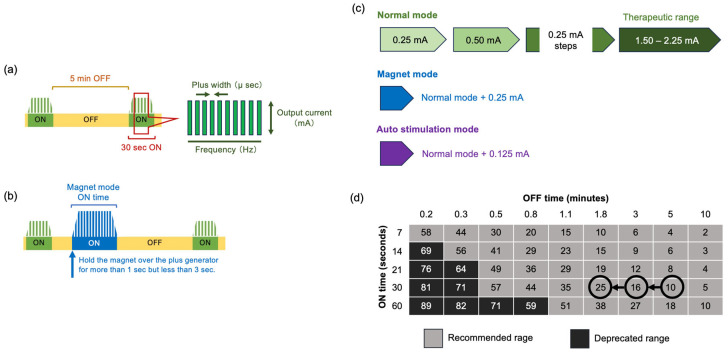
VNS parameter setting. (**a**) Basic VNS stimulation parameters: signal ON time, signal OFF time, plus frequency, plus width, and output current. (**b**) The magnet mode normally sets the output 0.25 mA higher than the normal mode. If patients feel an epileptic aura or their parents and guardians notice warning signs or repeated seizures within a short period, they hold the magnet over the plus generator for >1 s but <3 s. (**c**) Normal mode: 0.25 mA steps up to the therapeutic effect. Magnet mode: normal mode + 0.25 mA magnet mode. Autostim mode: normal mode + 0.125 mA. (**d**) Duty cycle: Increase the duty cycle over time and assess clinical outcomes. Adjustments to the duty cycle should be less frequent (3–6 months).

According to the research at the beginning of VNS therapy, two pivotal trials indicated that device settings of 30 s on/5 min off are safe and effective [14,15]. After VNS implantation surgery, stimulation is started at a low intensity and gradually increased while paying attention to the appearance of side effects. In the normal mode, it starts with an output current of 0.25 mA, a signal frequency of 20 Hz, a pulse width of 250 µs, a signal ON time of 30 s, and a signal OFF time of 5 min. It gradually increases while the effect on seizures is observed, as long as no adverse effects appear. If there is no effect, the duty cycle increases the ON time proportion in one cycle. The optimal conditions vary depending on the patient and require trial and error to determine in each case [16] (Figure 3a,c,d). In a recent study to identify an appropriate target dose for VNS therapy in epilepsy, the target output current and duty cycle were identified as 1.61 mA and 17.1%, respectively [17].

The magnet mode normally sets the output 0.25 mA higher than the normal mode. If patients are adults and feel an epileptic aura, they can start the magnet mode by placing it directly above the pulse generator for >1 s but <3 s. Even children with developmental delays may use the magnet mode when their parents and guardians notice warning signs or when they have repeated seizures within a short period. (Figure 3b,c).

## 5. Seizure Suppression Effect

**Figure 4:**
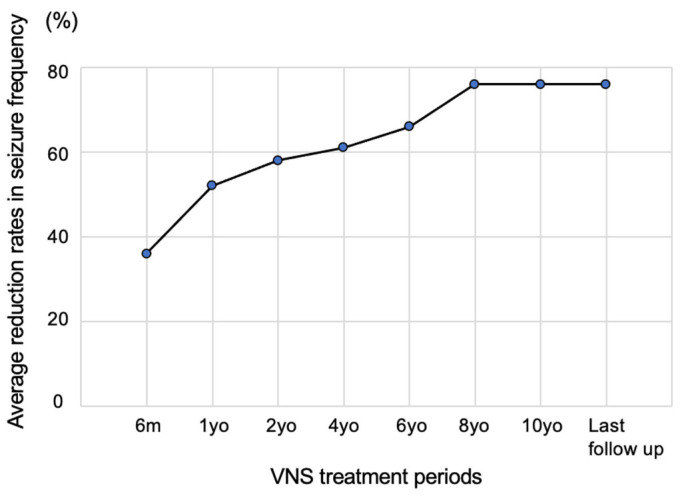
Seizure suppression effect. A study that followed 44 adults and 21 children over a long period reported that the mean reductions in seizures at 6 months and 1, 2, 4, 6, 8, and 10 years were 35.7, 52.1, 58.3, 60.4, 65.7, 75.5, and 75.5%, respectively. The seizure control rate increased slowly. Figure 4 was created by the authors based on the research results of Elliot et al. [20].

VNS reduces and improves seizure frequency over time. The McHugh classification has five levels of seizure frequency reduction: 80–100% (class I), 50–79% (class II), less than 50% (class III), only the effect of magnet use (class IV), and no effect (class V). The representative indicator using the McHugh classification is the 50% responder rate (the rate of patients whose seizures have been reduced by ≥50%, corresponding to classes 1 and 2) [18]. In a large-scale study focusing on adult epilepsy in the early stages of VNS initiation, the average seizure control rate was 20% at 3 months, 35% at 1 year, and 45% at 2 years after initiating stimulation. Furthermore, the 50% responder rate was 23% at 3 months, 37% at 1 year, and 44% at 2 years after initiating stimulation, confirming that seizures decreased over time [19]. Another study that followed 44 adults and 21 children over a long period reported that the 50% responder rate gradually increased and reached approximately 75% (Figure 4) [20]. Furthermore, a recent meta-analysis study indicated that seizure reduction (50% responder rate) for the VNS devices at years one, two, and three were VNS 32.9%, 44.4%, and 53.5%, respectively [21]. As described below, the efficacy and safety reported for children are approximately the same as those for adults. Conventionally, the Engel classification has been used to evaluate seizure improvement after epilepsy surgery; however, the treatment outcome classification proposed by McHugh et al. is often used to determine the seizure improvement effect after VNS [18].

## 6. VNS Device with an Automatic Seizure Control System Based on Heart Rate Fluctuations

This automatic seizure control function was included in the previous generation model, AspireSR (Figure 1a), and the current model, the SenTiva model manufactured by LivaNova, PLC in the London, UK, which has self-adjustment and automatic seizure adjustment functions based on heart rate fluctuations (Figure 1b). This model has been approved for use in the United States, Europe, China (from 2023), and Japan. The automatic seizure adjustment function identifies sudden tachycardia immediately before or during a seizure, identifies it as a seizure, automatically applies additional stimulation, and is expected to have a greater seizure suppression effect. As an effect of AutoStim mode, tachycardia, in which the heart rate increases by more than 50% during an attack, is observed in only approximately 15% of seizures, and it is necessary to lower the threshold for a heart rate increase. It has been reported that this treatment not only suppresses seizures but also improves QOL in approximately one-third of patients [22].

If the patients have a cardiac arrhythmia the automatic stimulation feature is not suitable. This includes heart conditions or treatments that do not allow necessary changes in the patient’s heart rate, such as atrial fibrillation, pacemaker dependency, implantable defibrillator, or cardiac medications such as beta blockers [23].

## 7. Clinical Effects and Benefits Other Than Seizures

**Figure 5:**
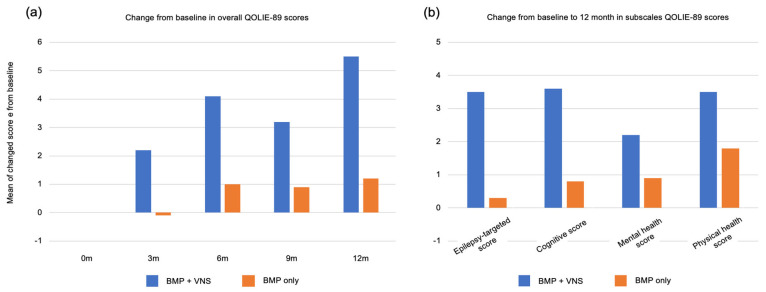
Clinical effects and benefits other than seizures: (**a**) Mean change in the Quality of Life in Epilepsy Inventory-89 (QOLIE-89) overall score from baseline (Month 0; n = 96) to months 3 (n = 94), 6 (n = 68), and 12 (n = 60). The QOLIE-89 score over time showed a significant difference between the VNS+BMP and BMP-only groups, with a greater improvement in patients allocated to the VNS+BMP group. (**b**) Change from baseline to month 12 in subscales QOLIE-89 scores. The differences were insignificant: epilepsy-targeted score (*p* = 0.06), cognitive (*p* = 0.20), mental health (*p* = 0.33), and physical health (*p* = 0.17). BMP: best medical practice, VNS: vagus nerve stimulation. Figure 5 was created by the authors based on the research results of Ryvlin et al. [25].

In addition to seizure suppression, various other clinical benefits have been reported. Reducing the number of hospitalizations, anti-seizure medications, and dosage is possible; therefore, it may be possible to reduce the side effects of anti-seizure medications. Moreover, a reduction in injuries due to a decrease in drop attacks and a decline in sudden unexpected death in epilepsy (SUDEP) due to a decrease in status attacks and a reduction in the burden on the cardiopulmonary system have been reported [24]. Furthermore, even in cases where seizures do not improve, improved emotional stability and sociability are often observed, especially by parents, resulting in positive outcomes for the child’s development and quality of life (QOL) (Figure 5) [25].

Furthermore, the 22 patients with DRE who underwent VNS were reported to have a significant decrease in seizures and interictal epileptiform discharges on their EEGs, as well as significantly improved sleep quality, compared to the control group [26].

## 8. Side Effects

**Figure 6:**
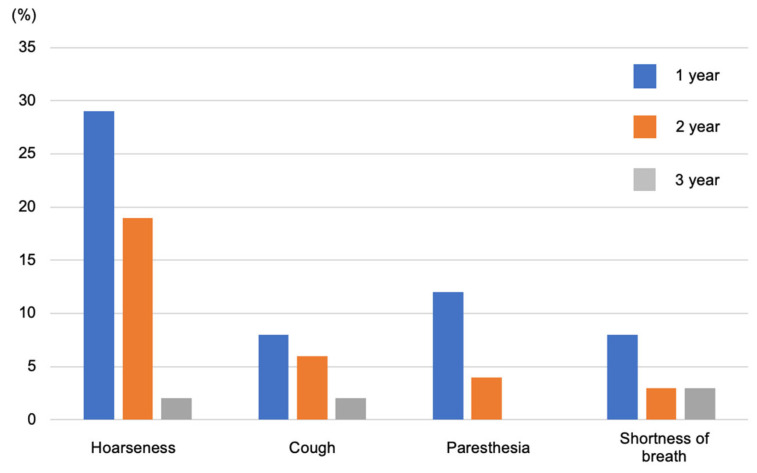
Side effects include voice changes, reactive coughing, and paresthesia pain in the back of the head, but these gradually improve. Figure 6 was created by the authors based on the research results of Morris et al. [28].

The most problematic side effect of VNS is wound infection. In a previous study of 808 patients, it was observed in 12 patients (1.5%), and the mean (±standard deviation) time from the most recent VNS-related surgeries to infection was 42 (±27) days [27]. Furthermore, side effects include reactive coughing, voice changes, paresthesia, pain in the back of the head, nausea, and salivation. However, they tend to subside over time and can be alleviated by changing the stimulation settings. Care must be taken in rare cases, as pain, scarring, and wound infection at the surgical site can sometimes cause irritation (Figure 6) [28,29].

## 9. Effect on Pediatric Drug-Resistant Epilepsy

Similar to adults, 70–80% of childhood epilepsy cases are benign and progress spontaneously. However, 20–30% of cases are resistant to anti-seizure medications, and persistent seizures and abnormal brain waves seriously affect a child’s development. VNS has certain suppressive effects on childhood DRE, the same as adults in the open-label multicenter study report of the analysis population, which included 347 children (aged 6 months to 17.9 years at the time of implant). It indicated that at 6, 12, and 24 months after implantation 32.5%, 37.6%, and 43.8% of patients, respectively, had a 50.0% responder rate in the baseline seizure frequency of the predominant seizure type. And, 19 patients (5.5%) were rendered seizure-free. A subset analysis using an age cut-off of <12 years at the initiation of VNS therapy demonstrated a 50% responder rate of 36.3%, 43.0%, and 50% at 6, 12, and 24 months, respectively, including 7.0%, 7.8%, and 11.3% of patients who were rendered free of the predominant seizure type. [30]. The efficacy rates in patients with childhood-onset epilepsy based on characteristic diseases are shown in Table 1.

Tuber sclerosis complex (TSC) is an autosomal dominant multisystem disorder that affects 1 in 6000 individuals [31]. Major et al. reported that the average age at VNS implantation was 15 years (range: 2–44; SD: 12.5), and the average duration of follow-up on VNS was 4 years (range: 0.5–8.6; SD: 2.3). The outcomes were rated as class I (>80% seizure frequency reduction) in three (19%), class II (50–79% reduction) in five (31%), class III (<50% reduction) in two (13%), class IV (magnet benefit only) in one (6%), and class V (no improvement) in five (31%) patients. Intermittent magnet use was effective in aborting seizures in eight patients (50%). Five patients (31%) reported improved functioning. Parain et al. reported that 10 patients with TSC with medically refractory epilepsy were treated using vagal nerve stimulation. Nine of them experienced at least a 50% reduction in seizure frequency, and five had a ≥90% reduction in seizure frequency. No adverse events were encountered [32,33].

Lennox–Gastaut syndrome (LGS) is a severe type of childhood epilepsy with an onset typically occurring before 8 years of age; it is almost always pharmacologically resistant. It has been reported that the 50% responder rate for LGS is >50%, and effects other than the seizure suppression mentioned above have also been confirmed [34,35]. In another report, a meta-analysis of 480 patients with LGS indicated that 54% of patients responded to adjunctive VNS therapy and that the treatment option was safe and well-tolerated [36].

Moreover, Dravet syndrome, a common childhood DRE, presents within the first year of life in a normal child with prolonged, febrile and afebrile, focal clonic (usually hemiclonic), or generalized clonic seizures. Seizures are usually intractable, and children demonstrate cognitive and behavioral impairments from the second year of life [37,38]. Recent studies about the effects of VNS on Dravet syndrome indicated that a more than 50% reduction in seizure frequency was observed in 36.4% (8/22), 54.5% (12/22), and 63.2% (12/19) of the patients at 12, 24, and 36 months, respectively [39], and another meta-analysis study indicated that 52.9% of Dravet syndrome patients experienced a ≥50% reduction in seizures [40].

*MECP2* mutations in female patients are the primary cause of Rett syndrome, which is a rare and severe X-linked neurodevelopmental disease caused by the gain of function of the *MECP2* gene. Seizures occur in 47% of the cases with *MECP2* mutations around 1 year of age, usually as part of a developmental and epileptic encephalopathy [41,42]. In a study of seven children with Rett syndrome who had DRE, five of the seven female patients had experienced at least a 50% reduction in seizure frequency at 3 months. At 12 months, six female patients had experienced at least a 50% reduction in seizure frequency. Four out of seven patients had at least a 90% reduction at 12 months. The two patients with 24 months of follow-up maintained more than a 90% reduction in seizure frequency from baseline [43].

Moreover, several studies have reported the effectiveness of DRE for childhood autism [44]. However, cases in which implantable devices cannot be tolerated also need to be considered. Regarding clinical effects other than seizures, a study of pediatric epileptic encephalopathy also reported that patients whose seizure frequency was reduced by at least 50% had significantly improved neuropsychological performance and QOL [45].

VNS is strictly a palliative treatment option for DRE. It is particularly recommended for cases in which the indications for epilepsy surgery are unknown, sufficient effects have not been obtained via surgery, or ketogenic diet therapy has not been effective. Symptomatic childhood epilepsy is the main target disease. However, for Dravet syndrome it is essential to first try anti-seizure medications such as Stiripentol and Fenfluramine hydrochloride [46]. Care must be taken not to include atypical self-remitting childhood epilepsy as an indication, such as self-limited epilepsy with centrotemporal spikes (SeLECTS).

## 10. Clinical Efficacy of the Latest Model SentTiva: An Illustrative Report

**Figure 7:**
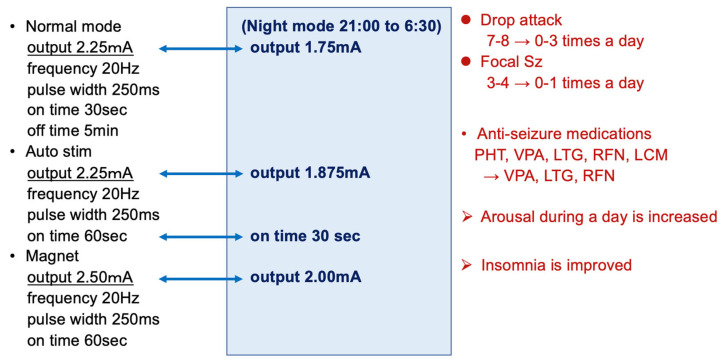
VNS parameter in a case study. As his seizures only occurred during the day when he was awake, we used the day/night mode to lower the stimulation intensity during sleep. VNS suppressed seizures by approximately 80%; further, by reducing the dose of anti-seizure medications, VNS efficacy significantly improved daytime alertness and insomnia. PHT, phenytoin; VPA, valproic acid; LTG, lamotrigine; RFN, rufinamide; LCM, lacosamide.

An 8-year-old boy with autism spectrum disorder presented with a 5-year history of drug-resistant tonic and myoclonic seizures with a drop attack. Notably, many anti-seizure medications could not suppress the seizures, and he was diagnosed with Lennox–Gastaut syndrome based on the seizure type and EEG findings. He was referred to our hospital and underwent a total corpus callosotomy when he was 5 years old. However, the drop seizures were not suppressed, and VNS placement (SenTiva Model 1000) was performed when he was 7 years old. The number of seizures, especially drop attacks, was reduced by approximately 80% by adjusting the VNS stimulation, and the level of alertness was also improved using pharmacological therapy. As his seizures occurred only during the day when he was awake, we used the day/night mode to lower the stimulation intensity during sleep (Figure 7). Besides this day/night setting function, the SenTiva Model 1000 has a schedule programming function that allows patients to adjust their stimulation without visiting the hospital.

## 11. Future Perspectives

VNS is essentially a palliative therapy, and its main limitation is that it cannot entirely suppress seizures. However, there is potential for various improvements that may increase the utility of VNS. One is to identify biomarkers that indicate the efficacy of preoperative VNS. There are reports that predict the effects of VNS using resting-state magnetoencephalography (rMEG) and resting-state functional MRI [47,48]. In addition, the effects of VNS can be predicted via the analysis of scalp EEGs, which are commonly performed, but this prediction method may be more difficult [49,50,51]. The second is to clarify the seizure types (e.g., focal vs. generalized) and epilepsy syndromes for which VNS is effective and to clarify the optimal dose setting for each. Furthermore, it was reported that the use of VNS earlier during the course of epilepsy was significantly related to responder status and QOL improvement [52,53]. However, there are still too few clinical trials of prognostic factors for VNS therapy in children. Future studies should aim to address these questions and improve the technology used in VNS to further expand the role of VNS therapy for DRE in children.

## 12. Conclusions

VNS affects seizure control; however, other benefits, such as improved sleep quality and emotional stability, fewer hospitalizations, and the possibility of reducing or discontinuing medication, have been reported. The VNS stimulation intensity can be adjusted while monitoring seizure suppression and side effects. VNS treatment is useful when combined with anti-seizure medications, as the stimulation intensity can be changed during the day and night, and additional stimulation can be performed during or just before seizures. VNS is extremely useful because it positively affects the development and QOL of children with DRE.

## Figures and Tables

**Table 1:** Efficacy of VNS for childhood DRE.

Underlying Disease	Typical Age of Epilepsy Onset	50% Responder Rate
Tuberous sclerosis	<1 year old [31] (1 year old on average)	50–90% [32,33]
Lennox–Gastaut syndrome	Median age 4 years [34]	50–65% [34,35,36]
Dravet syndrome	<1 year old [37,38]	36–63% [39,40]
Rett syndrome (MECP2 genetic mutation)	6–18 months old [41,42]	86% [43]

## Data Availability

Not applicable.
